# Supplementary material for: Empirical methods for controlling false positives and estimating confidence in ChIP-Seq peaks
Source: BMC Bioinformatics. 2008 Dec 5;9:523. doi: 10.1186/1471-2105-9-523 (PMC2628906; doi:10.1186/1471-2105-9-523)
Supplement: Additional File 1 — A variety of html documents from the USeq web site detailing the available applications, their best usage, output file type descriptions, command line menus, etc. [file 1471-2105-9-523-S1.zip › USeqUserGuides/JavaDoc.zip!/JavaDoc/bioroot/package-frame.html]

bioroot


bioroot

Classes 
  
AccountManagement
  
Archive
  
BlastMatcher
  
CellType
  
CellTypeBean
  
Contact
  
ContactBean
  
CronArchive
  
DBUtil
  
DeleteMeNow
  
Feature
  
Gene
  
GeneBean
  
IdNameMatch
  
LabGroup
  
Login
  
LoginBean
  
Logout
  
Marker
  
MarkerBean
  
Organism
  
OrganismBean
  
Organization
  
PatternMatch
  
Preference
  
ReagentBean
  
Search
  
SearchBean
  
SearchHit
  
Spammer
  
TableHeading
  
UserBean
  
UserRegistration
  
UserRegistrationBean
  
Util
